# Migraine and Meat

**Published:** 1905-04-08

**Authors:** 


### MIGRAINE AND MEAT.

In a letter to a contemporary Dr. Alexander Haig[1] again comes forward as a champion of the uric acid origin of migraine. He says that in a series of between 500 and 1,000 cases—a somewhat wide margin of error to allow his memory—success attended his dietetic treatment in not less than 75 per cent. He proceeds to add that he could count on the fingers of one hand the failures following a prolonged and honest fulfilment of his instructions. It is difficult to see the advantage of confining the labour to one hand when 125 cases have to be dealt with, assuming the lowest computation.

The uric acid bogey is being gradually exposed, and we suspect that there are not a great number of practitioners who believe as honestly as Dr. Haig in the universal malignity of uric acid. This is not to deny the great benefits often derived from a careful limitation of meat in the dietary of those out of health from many causes. There is no room for doubt that in our sedentary existences the habitual consumption of meat exceeds the limits of prudence, and that after middle age particularly, a restriction of animal proteids is often followed by undeniable betterment of health. The truth is that it is harder to over-eat one's self on a vegetarian than on a meat diet, partly because the labour involved is greater, partly because vegetables are not so sapid and tempting to the palate as those containing meat-juices. Moreover, Pawlow has shown that the contact of meat-juices with the mouth provokes a secretion of the gastric juice incomparably greater, for a time, than that following the ingestion of vegetables or milk.

Dr. Haig's letter will convince no sceptics, least of all those who bear in mind the natural tendency of migraine to diminish in its frequency and severity as age advances.

[1] Lancet, March 25, 1905.

